# Hydraulically Coupled Dielectric Elastomer Actuators for a Bioinspired Suction Cup

**DOI:** 10.3390/polym13203481

**Published:** 2021-10-11

**Authors:** Chi Zhang, Lei Liu, Kanghui Xu, Zhonghong Dong, Yuxi Ding, Qi Li, Pengfei Li

**Affiliations:** 1 Key Laboratory of Expressway Construction Machinery of Shaanxi Province, Chang’an University, Xi’an 710064, China; xjtuzc@gmail.com (C.Z.); dzhong@chd.edu.cn (Z.D.); 2 School of Mechanical and Precision Instrument Engineering, Xi’an University of Technology, Xi’an 710048, China; kanghuixu142@163.com (K.X.); dingyuxi1103@163.com (Y.D.); lipengfeinew@xaut.edu.cn (P.L.); 3 Highway School, Chang’an University, Xi’an 710064, China; liqi@chd.edu.cn

**Keywords:** dielectric elastomer, suction cup, bioinspired, electrically zipping, hydraulically coupled

## Abstract

Suction cups of cephalopods show a preeminent performance when absorbing irregular or flat objects. In this paper, an octopi-inspired suction cup, driven by hydraulically coupled dielectric elastomer actuators (HCDEAs), is proposed, which is considered to be controlled easily and have compact structure. To investigate the performance of suction cups, experiments have been conducted to clarify the effect of the pre-stretch ratio and chamber angle on suction forces. It could be seen that both factors have a complicated influence on suction forces, and the best performance obtained was a reasonable combination of the pre-stretch ratio and chamber angle. Here, we achieved a maximum suction force of 175 mN with λ_p_ = 1.2, α = 23° under a DC voltage of 3500 V. To enhance the capacity and adaptation of the suction cup, flat objects of various types of materials were introduced as targets. Experimental results displayed that for tested materials, including a dry/wet acrylic plate, CD, ceramic wafer, and aluminum plate, the suction cup showed outstanding performance of absorbing and lifting the target without any damage or scratch to them. Our research may serve as a guide to the optimal design and provide insights into the performance of the HCDEAs-actuated suction cup.

## 1. Introduction

In robotics, the ability of robots to grasp and manipulate fragile and irregular objects is of great concern, especially when the target is flat and easy to scratch. Dexterous cephalopods with soft arms and distributed suction cups provide bionic solutions since they can adsorb and release wet and dry objects in different sizes and shapes with ease [1,2,3,4]. In recent years, many researchers have been devoted to designing and fabricating novel bio-inspired suction cups [5,6,7,8,9,10,11,12,13,14,15,16]. Nguyen et al. [5] fabricated an adhesive pad covered by a distributed passive suction cups array, which showed a three-times higher suction force than the theoretical limit, available for both dry and wet surfaces. Inspired by protuberances in the suction cups of octopuses, Baik et al. [7] designed an adhesive patch fully covered with micro suction cups by air trapped technology, which functioned well on human skin with hair and sweat. To actively control the adsorption and release of suction cups, Satoshi Nishita and Hiroaki Onoe [8] used liquid to inflate and deflate a fully polymeric suction cups array, consequently realizing fast attachment and detachment. To grasp objects of irregular shapes and in confined environments, Mazzolai et al. [9] designed and fabricated a soft arm mimicking octopuses’ tentacles, which consists of an elastic arm made by polydimethylsiloxane and a pneumatically actuated suction cup array. Besides the conventional passive and fluid actuated suction cups, some researchers attempted to employ new actuation mechanisms [11,15,16,17]. Lee et al. [11] fabricated micro suction cups, of which the inner chamber is covered by temperature-sensitive hydrogel, changing the chamber volume under different temperatures and reversely switching the adhesive force. Hu [15] introduced a shape-memory-alloy spring to control the suction cup chamber volume, which could be adjusted by the heating or cooling of an electric coil. Wang et al. [16] employed magnetically sensitive materials to change the volume of a suction cup chamber, which achieved a fast-response adhesion and high energy efficiency, within a comparably compact size.

As described above, tremendous advances have been achieved by researchers in many aspects, including actuation resources, suction cup structure and fabrication technology. Nevertheless, inherent problems still exist, greatly hindering the application and commercialization of bioinspired suction cups. Passive suction cups are low-cost, easily fabricated and well-adapted for both wet and dry surfaces. However, active detachment with objects has failed to be achieved. For suction cups actuated by an external pneumatic compressor or fluid pump, the bulky structure makes it tricky in regards to miniaturization and being lightweight. Shape memory alloys and temperature-sensitive hydrogels are comparably lightweight and compact; however, the accurate and fast control of temperature is challenging and unpredictable considering the complicated application surroundings. Magnetic-actuated suction cups feature rapid absorption and release with a considerable adhesive force. The employment of an electromagnet, on the other hand, means high current and heat dissipation issues. In short, a novel actuation mechanism for suction cups is desired, which should be easy-controlled, fast in its response, compact and lightweight, safe in use, etc.

Over decades, the emergence and development of electroactive polymers has provided powerful solutions for efficient, compact and smart actuation. Electroactive polymers, also known as EAPs, are normally polymers which respond to stimuli and show an outstanding deformation or force output. The generally used EAPs include IPMC (Ionic Polymer/Metal Composites), DEs (dielectric elastomers), CPs (conducting polymers) and many other kinds of materials, all of which could be categorized into two types, namely dielectric and ionic types [18,19,20,21]. As a sub-category of electro-active polymers, dielectric elastomers (DEs) are considered as promising artificial muscles for their outstanding features, such as large strain, low cost, high energy efficiency and fast response [22,23,24,25], and thy have been applied in many fields, such as as biomimetic robots [26,27], energy harvesters [28,29] and various actuators [30,31,32], since they were first reported by Pelrine [18]. DEs achieved great progress when Keplinger et al. [33] presented HASEL (hydraulically amplified self-healing electrostatic) actuators, which showed better performances than traditional DEs with a larger actuation strain, lower risk of breakdown, longer lifespan and self-healing ability. After that, HASEL actuators with a linear contraction [34] and their improved version [35] were reported in sequence, both exhibiting excellent properties as linear soft actuators. Besides this, Leroy et al. [36] explored micro HASEL actuators at the microns level and fabricated an actuator with high permittivity materials. In sum, the employment of dielectric liquid for DEs led to more outstanding performances and was more favorable for compact actuators.

Herein, based on the excellent properties of HASEL actuators, we designed and fabricated hydraulically coupled dielectric elastomer actuators (HCDEAs) and applied them to drive suction cups. The effects of pre-stretch ratio and chamber angle on the suction force were investigated. The absorption function of suction cups on materials with different surface characteristics was also explored. The remainder of this paper is organized as follows. Section 2 describes the design and fabrication process of the suction cups and the experimental setup for suction force measurement. Experimental results are shown in Section 3, and the feasibility of suction cups on flat objects of different materials are also displayed. Conclusions of the article and prospects of the project are detailed in Section 4.

## 2. Experimental

### 2.1. Design of Prototype

**Figure 1:**
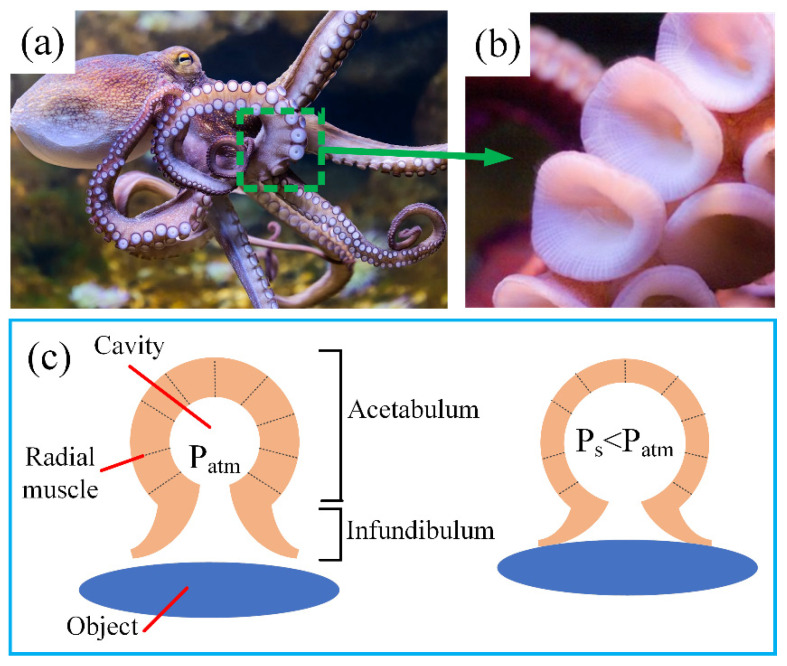
Working principle of the suction cups of octopuses. (**a**) The tentacles of an octopus are covered by distributed suction cups; (**b**) enlarged view of arrayed suction cups; (**c**) a pressure differential is created by the contraction of radial muscles.

As shown in Figure 1a,b, cephalopods, especially octopuses, show an outstanding capability of grasping objects of various sizes and shapes due to their totally soft tentacles and manipulating delicate and small objects, owing to their ingenious suction cups that are well-distributed along their arms [37]. According to investigations of Tramacere et al. [38], the anatomical structure and attachment process of individual suction cups are quite complicated, which is the combined result of several kinds of muscles, and the absorption process is divided into several stages. In this article, we employed a simplified model to illustrate the working mechanism of individual suction cups, which is considered reasonable [2]. As shown in Figure 1c, a suction cup mainly consists of two parts, namely the acetabulum and infundibulum. The former has a spherical chamber, which changes volume in different working stages, while the latter makes contact with the object directly, forming a sealed chamber. When the suction cup absorbs the object, the radial muscles denoted by the black dotted lines contract along the wall thickness direction, consequently decreasing the chamber pressure dramatically, forming a pressure differential and adhering to the object tightly.

**Figure 2:**
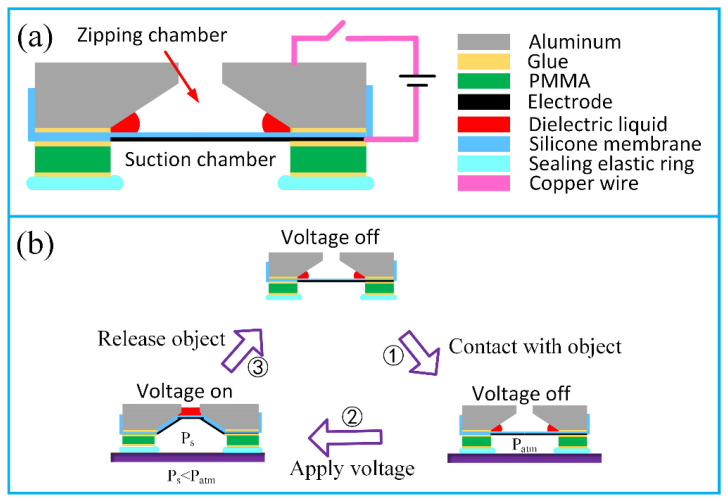
Structure and working process of the suction cup actuated by HCDEAs. (**a**) Cross-section view of the HCDEAs-actuated suction cup; (**b**) Voltage-controlled working process of the HCDEAs-actuated suction cup.

The aim of this paper was to design an octopus-inspired suction cup, which can absorb objects by mimicking the contraction of radial muscles. Based on the outstanding properties of HASEL actuators, we combined a dielectric liquid with the electrostatic zipping phenomenon and proposed hydraulically coupled dielectric elastomer actuators (HCDEAs); then, we employed HCDEAs to actuate a bio-inspired suction cup. Figure 2a shows the cross-sectional view of the suction cup we developed, which mainly consists of three parts. The mechanically actuated part is an HCDEA composed of an elastic membrane (Sylgard 186, Dow Corning, Midland, MI, USA) under equi-biaxial pre-stretch and an aluminum part, which was milled to form a conical chamber. The pre-stretched membrane and the aluminum part were bonded together by PET-based adhesive tape. Conductive rubber (EL-8006-6, ADHESIVE RESEARCH, Glen Rock, PA, USA) with good conductivity, even during a big stretch, was employed here to work as an elastic electrode, and it was leaded by copper foil. To augment the electrostatic zipping force between the elastic electrode and aluminum chamber wall, dielectric liquid FC-40 (3M Company, Sao Paulo, MN, USA) was introduced here for its high permittivity and stability. Subject to voltage, charges with opposite polarities accumulated on the elastic electrode and aluminum part, respectively, and attracted each other, inducing the membrane to “collapse” upward. The second part is a PMMA circular ring, bonded to the bottom of an elastic membrane by the adhesive tape, forming a chamber similar to the octopus’s suction cup. At the bottom of the device is a casted silicone ring (ECOFLEX 0010, SMOOTH ON, Macungie, PA, USA), which functions as the sealing part, since ECOFLEX 0010 is considerably soft with a low modulus.

Figure 2b illustrates the working process of a suction cup which is a closed loop consisting of three steps. At the first step, the suction cup makes contact with the object surface, forming an enclosed space with the aid of the sealing ring. A voltage is applied to the elastic electrode and aluminum part, driving the elastic membrane to overcome the rebound force and negative pressure to deform upward. The enlarging of the suction chamber’s volume causes a pressure decrease inside it, consequently bonding the device to the object. At the last step, the voltage is removed, and the elastic membrane deforms to its original state due to rebound force and outside air pressure; as a result, the object is released.

### 2.2. Fabrication of the Suction Cup

**Figure 3:**
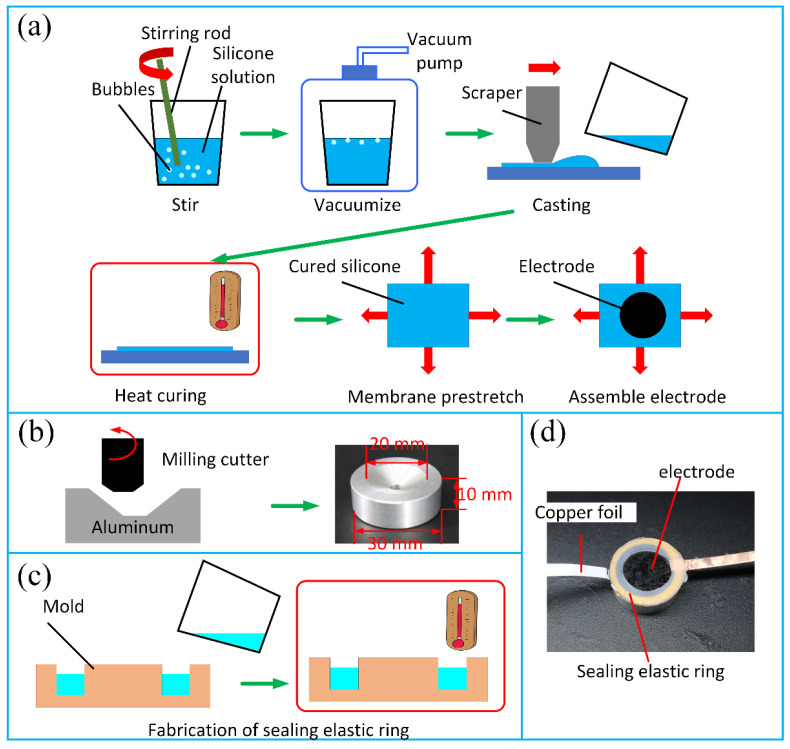
Fabrication process of the HCDEAs-actuated suction cup. (**a**) Preparation of a pre-stretched Sylgard 186 membrane coved by elastic electrodes; (**b**) aluminum part processing; (**c**) casting of the sealing elastic ring with Ecoflex 0010 solution; (**d**) prototype of the HCDEAs-actuated suction cup.

It can be seen from Section 2.1 that the suction cup mainly consists of three parts, which are in a stacked-up form and bonded by adhesive tape. In this section, the whole fabrication process of the suction cup is divided into four steps and detailed. As shown in Figure 3a, the first step is the preparation of the elastic membrane, which is basically referring to the standard silicone casting process developed by Rosset et al. [39]. The elastic membrane solution used here was Sylgard 186, which has a mixed viscosity of 66,700 cP. The silicone solvent OS-20 (Wacker Chemie AG, Munich, Germany), with a mass fraction of 37.7%, was added to the silicone solution to decrease the viscosity, which was too high to cast. After vacuumizing for around 10 min, the mixture (62.3% Sylgard186 and 37.7% OS-20) was casted on a 125 μm-thick PET substrate by an applicator (PF200-H, Lebo Science, Jiangyin, China) with a 200 μm gap and 10 mm s^−1^ casting speed. The casted solution was then placed in an oven carefully for 60 min at 80 °C, and a cured silicone membrane was produced with an average thickness around 55 μm. The cured membrane was then pre-stretched and fixed by a PMMA frame while a laser cutting conductive rubber was bonded to the membrane as the elastic electrode. The aluminum part with a conical chamber was milled by CNC, and a through-hole with a diameter of 4 mm was drilled for the dielectric liquid infusion. The sealing elastic ring was casted by an inverse mold, forming a circular ring with an inner and outer diameter of 20 and 30 mm, respectively, while the thickness was 2 mm. The prototype of the suction cup after assembly is shown in Figure 3d, with two copper foils as electrical connections.

### 2.3. Experimental Setup and Procedures

**Figure 4:**
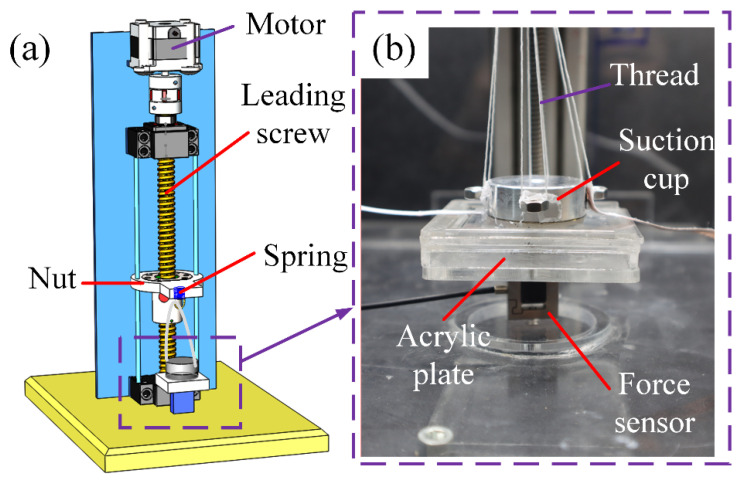
Schematics of the experimental setup for suction force measurement. (**a**) A 3D rendering of the experimental setup; (**b**) enlarged view of the attachment between the suction cup and acrylic plate.

The main performance index of suction cups is the suction force between the device and the object surface with different characteristics. Herein, a test-bench was set up to measure the suction force, as shown in Figure 4. The test-bench consisted of a motorized linear stage, which was used to drive the nut running along the leading screw and a force sensor (SBT630-2N, Simbatouch, Guangzhou, China) to measure and record the stretching force. The force senor was fixed to the base of the stage by a screw, and the other end was fixed to an acrylic platform, which had a slot to fix the acrylic plate for suction. The suction cups were connected to the sliding nut by four threads and a spring. The employment of a spring here was to reduce the rate of the stretching force increasing. During testing, the suction cups were powered by a high voltage supply (Model 610E, Trek, New York, NY, USA), and the voltage signal was generated by a signal generator (DG4062, Rigol, Suzhou, China). The procedures for the experiments were arranged as follows:

1. Place a clean acrylic plate on the platform slot, and place the suction cup on it while the other end of suction cup is connected to the threads.
2. Connect the force sensor to a laptop by a cable, and turn it on. Set a DC voltage by a signal generator and power the high voltage supply on.
3. Turn on the motor, and record the data of the force sensor using the laptop.
4. Power off all the devices once the suction cup detaches from the acrylic plate. The maximum value of the recorded curve is the maximum suction force.

## 3. Results

### 3.1. Effect of Pre-Stretch and Chamber Angle on Suction Force

**Figure 5:**
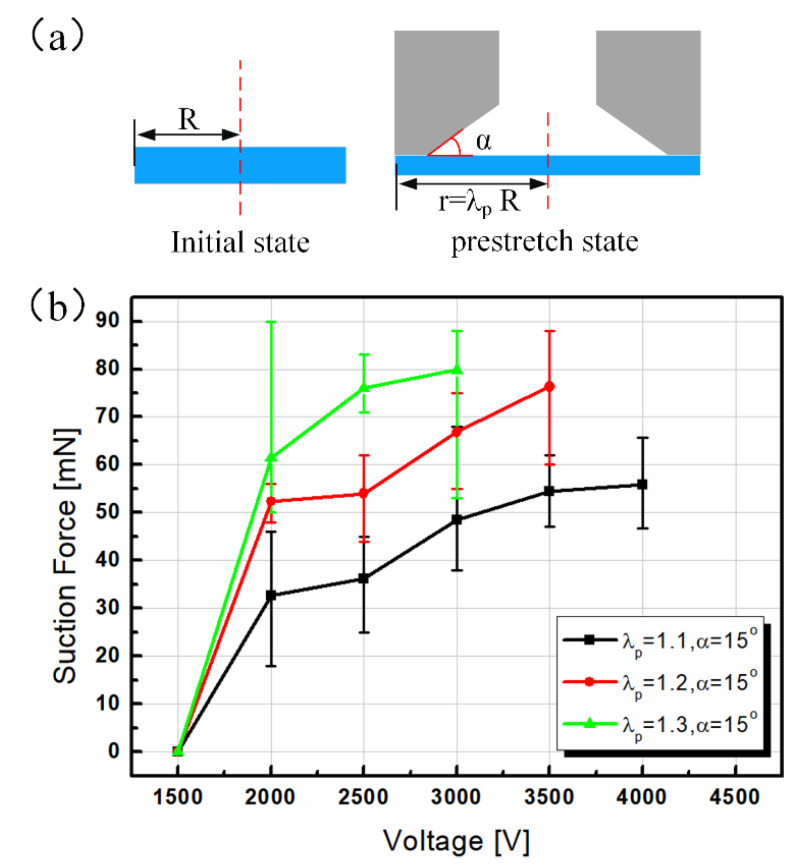
Experimental results of suction force measurement. (**a**) Sketch map of the pre-stretch ratio and chamber angle; (**b**) relation between the suction force and voltage under different pre-stretch values and the same chamber angle.

It can be seen from Section 2 that many factors affect the final performance of suction cups, including both geometric parameters and employed materials’ properties. Investigating the effects of all relevant factors requires tremendous efforts and is impractical. Herein, we focused on two principal factors, namely the pre-stretch ratio and chamber angle, which have been proven as the dominating factors of zipping dielectric elastomer actuators [36,40]. As shown in Figure 5a, the pre-stretch ratio is defined by $\lambda_{p}=r/R$, where *r* and *R* represent the radius of the circular elastic membrane in the pre-stretch and initial state, respectively. The chamber angle is denoted by α, which represents the angle between the pre-stretched membrane and the sidewall of the zipping chamber. Another factor is the dielectric liquid, which is thought to mainly have effects on the dynamic response of the suction cup [34,36]. In this paper, we used FC-40 and ensured that the infused mass of liquid for every test was equal. All of the following experiments were conducted with the experimental setup shown in Figure 4.

Figure 5b plots the relations between the suction force and the applied voltage at three given pre-stretch values, $\lambda_{p}=1.1,\;1.2,\;1.3$, while the chamber angle was fixed to 15°. For every sample, the voltage increased from 500 V with a 500 V step until the suction cup reached electrical breakdown. Since no obvious results were observed when the voltage was lower than 1500 V, the axis denoting voltage started from the value of 1500 V. With the increase of voltage, all three curves showed an abrupt rising trend when the voltage ranged from 1500 to 2000 V, which means that the zipping phenomenon occurred [40]. When the voltage continued to increase, the slope of the three curves diminished gradually, which signifies that the promoting effect of voltage on suction force was weakened. It was noted that under the same voltage, the suction force increased with the enlargement of the pre-stretch. This phenomenon was interpreted to indicate that as a higher pre-stretch ratio means a thinner elastic membrane, consequently, a higher actuation electric field was obtained. Although a higher pre-stretch also means that the elastic membrane is more difficult to deform, since it possesses more elastic energy, the promotion of an electric field surpasses the resistance of elastic energy, which ultimately leads to a bigger suction force.

**Figure 6:**
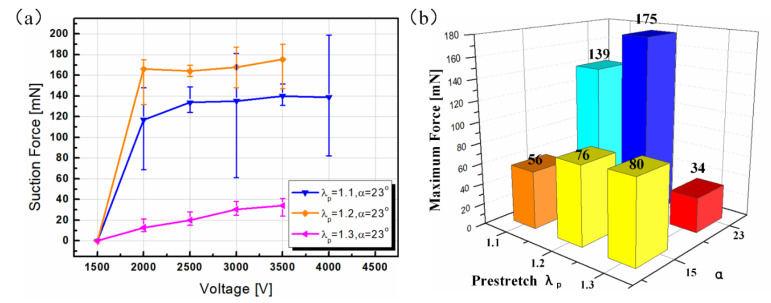
Experimental results of suction force measurement. (**a**) The relation between suction force and voltage under different chamber angles and the same pre-stretch; (**b**) the effect of the pre-stretch ratio and chamber angle on the maximum suction force.

Nevertheless, when the chamber angle enlarged to 23°, the effect of pre-stretch on suction force under the same voltage was totally different from the condition with $\alpha =15^{{}^{\circ}}$, as shown in Figure 6a. For samples with $\lambda_{p}=1.1,\;1.2$, the curves of the suction force displayed an abrupt rise when the voltage boosted to 2000 V, after which the curves became roughly flat, implying that the voltage has a limited promotion of the suction force. This phenomenon makes a lot of sense because we can employ an actuation voltage far away from the critical voltage in practical applications to guarantee safety and stability. Compared with the curves in Figure 5b, samples with $\lambda_{p}=1.1,\;1.2,\;\alpha =23^{{}^{\circ}}$ showed a bigger suction force than prototypes with $\lambda_{p}=1.1,\;1.2,\;\alpha =15^{{}^{\circ}}$ under the same voltage. This result is explained, as a larger chamber angle means more volume change of the suction chamber; consequently, a bigger pressure differential is generated, along with a bigger suction force. Further enlarging the pre-stretch ratio to 1.3, the curve showed an approximately linear trend with a small slope. In sum, under $\alpha =23^{{}^{\circ}}$, the effect of pre-stretch on suction force was nonmonotonic under a given voltage. To systematically understand the effect of chamber angle on the performance of a suction cup, we continued to enlarge the chamber angle to 32°. However, no obvious suction force was measured, and all the samples with $\lambda_{p}=1.1,\;1.2,1.3$ showed no absorption until electrical breakdown occurred.

To display the effect of pre-stretch and chamber angle on the maximum suction force comprehensively, a three-dimensional diagram was plotted. Note that the experimental result with $\alpha =32^{{}^{\circ}}$ was not depicted since all data was zero. As shown in Figure 6b, at a low level of the chamber angle ($\alpha =15^{{}^{\circ}}$), the maximum suction force varied monotonically with the increase of the pre-stretch. On the contrary, the maximum suction force changed nonmonotonically at a moderate level of the chamber angle ($\alpha =23^{{}^{\circ}}$). Compared to the sample with $\lambda_{p}=1.2,\;\alpha =23^{{}^{\circ}}$, the maximum suction force of the sample with $\lambda_{p}=1.3,\;\alpha =23^{{}^{\circ}}$ showed a dramatically decrease. This phenomenon was clarified, as enlarging the prestretch to a higher level means a higher electric field induced for the samples under the same voltage. However, a bigger pre-stretch also signifies that the membrane is more detrimental to bend since it features higher resilience. The higher resilience, along with a bigger chamber angle ($\alpha =23^{{}^{\circ}}$), works as resistance against the membrane’s deformation, which functions more strongly than the promotion of a pre-stretch-induced higher electric field, consequently resulting in a huge decrease. At a high level of the chamber angle ($\alpha =32^{{}^{\circ}}$), the distance between the aluminum electrode and the elastic electrode was too big so that no zipping behavior could occur, no matter what the pre-stretch was and how big the voltage was. This experimental result is thought to be favorable for suction cups’ optimization.

### 3.2. Characterization of the Suction Cup and Array on Different Types of Surfaces

**Figure 7:**
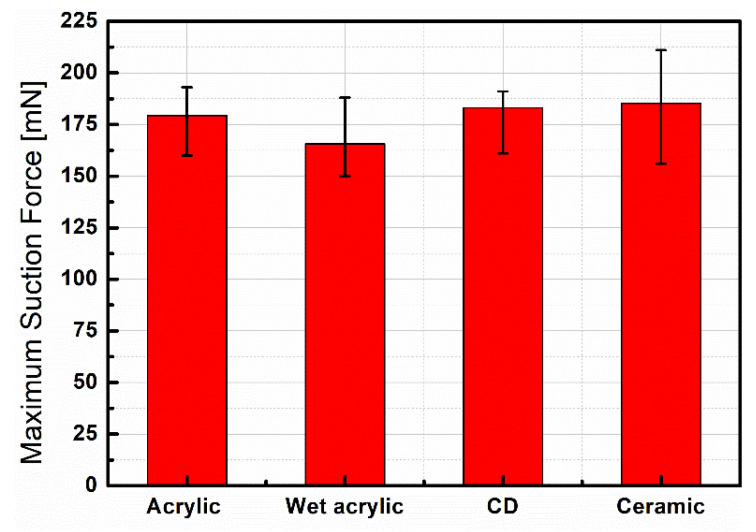
Measured maximum suction force of the device on different types of surfaces under 3500 V DC.

In this section, we investigated the versatility and utility of the suction cup by characterizing its performance on various surfaces and assembling four suction cups into a 2 × 2 array to improve their capability and adaptability. Based on the experimental results, the parameters selected here were $\lambda_{p}=1.2,\;\alpha =23^{{}^{\circ}}$, since suction cups have the best performance under these conditions, as shown in Figure 6b. The experiment was conducted with the test-bench and followed both the procedures presented in Section 2.3. In Figure 7, the maximum suction force of a single suction cup on different types of surfaces was measured and compared. The materials employed here were an acrylic plate in dry and wet conditions, CD and ceramic wafer. Under a voltage of 3500 V DC, the maximum suction force on all the materials was approximately equal, ranging from 165 to 180 mN, which means that our device has the potential for the manipulation of the tested materials.

**Figure 8:**
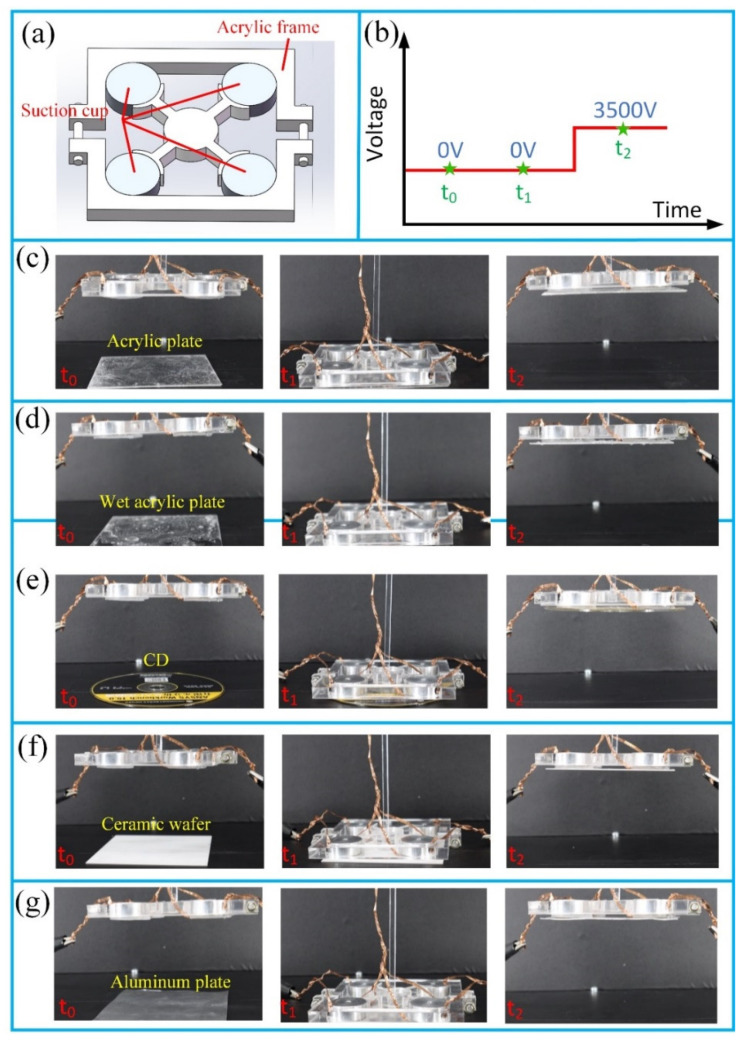
Demonstration of the suction cup array’s wide capability of absorbing flat objects of different materials. (**a**) Sketch of the 2 × 2 suction cup array; (**b**) waveform of the applied voltage; (**c**) acrylic plate; (**d**) wet acrylic plate; (**e**) CD; (**f**) ceramic wafer; (**g**) aluminum plate.

A single suction cup has a limited adaptability and load capacity in confined environments. Therefore, they are normally installed onto soft artificial arms in a specific configuration [9]. Here, we designed an acrylic frame for suction cups’ installation, and the assembled device was tested for its ability to manipulate flat objects which were fragile and easy to scratch. As shown in Figure 8a, four suction cups were arrayed in a 2 × 2 configuration by the acrylic frame. The applied voltage was 3500 V DC, and three moments were selected, with the snapshots displayed in Figure 8c–g. It can be seen that all the objects wre grasped and lifted by the suction cup array without any damage, which is also shown in Video S1. A potential application of our device is to grab fragile and delicate flat objects, which is really tough work for traditional rigid mechanical arms.

## 4. Conclusions

In this article, an octopus-inspired suction cup, actuated by hydraulically coupled dielectric elastomer actuators, was designed and fabricated. To characterize the performance of the suction cup, a test-bench was constructed, and two main factors, including the pre-stretch ratio and chamber angle, were experimentally investigated. The results revealed complicated relations between the suction force and corresponding factors. At a low level of the chamber angle, enlarging the pre-stretch ratio enhanced the performance of the suction cup, while the suction force showed a nonmonotonically changing trend at a moderate chamber angle. The suction cup, however, showed no absorption function when the chamber angle was too big. For our device, a maximum suction force of around 175 mN was achieved at $\lambda_{p}=1.2,\;\alpha =23^{{}^{\circ}}$ under 3500 V DC. The studies carried out here provide the design guidelines for an HCDEAs-actuated suction cup to achieve the best absorption performance. We further studied the versatility and utility of the designed suction cup by testing its function on various types of materials, which showed an excellent performance in absorbing and lifting flat objects without damaging or scratching the target. In sum, the suction cup we fabricated in this paper is of a compact size, easy to control by voltage and has no requirement for an external compressor, which makes it a favorable candidate for soft and mechanical robotic arms for soft manipulation.

## Data Availability

The data presented in this study are available on request from the corresponding author.

## Supplementary Materials

The following are available online at https://www.mdpi.com/article/10.3390/polym13203481/s1. Video S1: Absorbing and lifting flat objects of various materials by the suction cup array.
